# The Potential Impact of Reliance on Expressed Milk Feeding for Maternal and Child Health

**DOI:** 10.3390/children3040025

**Published:** 2016-11-03

**Authors:** Genevieve Becker

**Affiliations:** BEST—Breastfeeding Education Support and Training—Services, 2 Kylemore Park, Taylor’s Hill, Galway, Ireland H91T22T; gbecker@bestservices.ie

Human milk has nourished human babies for thousands of years and its importance is widely recognised. Increasingly, babies are receiving human milk as expressed or pumped milk and not at the breast, while the impact of this change is still unknown. The method of expressing or pumping milk can result in differences in nutritional content of the milk, such as protein, sodium and fat content, which has particular relevance to infants in neonatal units, who rely solely on this milk [1]. Additionally, immunological properties may differ between milk suckled directly from the breast and milk expressed, stored and warmed before feeding, although this difference does not appear to have been thoroughly studied.

When feeding at the breast, the baby’s oral movements compress the breast in a rhythmic motion, which creates a changing pressure gradient assisting the flow of milk. The baby’s suckling also stimulates the release of the maternal hormone oxytocin, which contracts the myoepithelial cells that surround the milk producing cells and propels milk along the ducts towards the nipple. Expression relies on assisting the release of oxytocin and compression of the milk ducts, whereas pumps generally rely on changing the pressure gradient between the pump and the breast. Hand expression of milk and pumping are physiologically different, however, terminology is often used interchangeably, thus limiting comparison of the two methods [1].

The control of feeding is also different, dependent upon the feeding technique. For instance, a baby removes milk from the breast by suckling and is able to control the amount of milk received, whereas a bottle feed is often entirely controlled by the caregiver. The article by Yourkavitch et al. published in this Special Issue [2] examines if the means of delivering the milk—by bottle or directly from the breast—is associated with the incidence of gastro-esophageal reflux in exclusively breast milk fed infants. Although a significant association was not found in the secondary analysis of a large USA data set, important questions are raised that are of interest to further research on the impact of how expressed milk is fed.

If pumping is becoming so common among new mothers, particularly in the USA, what information do mothers seek and where do mothers obtain information about pumping? Pump manufacturers and distributors may focus on marketing and increasing sales of their products, rather than providing independent, clear information. Many new mothers use online sources and Yamada et al. [3] explore the questions that are asked in an online discussion forum, and how these questions vary across time-points, from pregnancy, through the early weeks after birth and later.

The financial toll of purchasing equipment for pumping milk may often be discussed, however there is also an environmental cost to increasing reliance on pumped milk. In this Issue, Becker and Ryan-Fogarty [4] outline some of these environmental issues, including heavy metals in electronic pumps, waste plastics as pollutants and disposal concerns, as well as broader environmental concerns of increased obesity linked to bottle feeding and possible effects on the population as a whole.

Mothers express or pump milk for a wide variety of reasons, including a baby who is unable to suckle due to prematurity or abnormality, separation of mother and baby, or personal preference of the mother. Greater awareness and more research is needed of the potential impact for the child, the mother, and the wider society, of the use of expressed or pumped milk. This Special Issue hopes to serve as a step towards increasing that awareness.
